# Remarks on the Advance in Practice and Use of Mechanical Appliances

**Published:** 1874-12

**Authors:** M. Bissell


### REMARKS ON THE ADVANCE IN PRACTICE AND USE OF MECHANICAL APPLIANCES.

BY M. BISSELL.

The present may be characterized as decidedly an age of improvement, for the past thirty years have been replete with progressive developments in all departments. In the invention of the electric telegraph, the discovery of photography, in the construction of railways, the navitation of the ocean by steam, the application of chemistry to physiology, leading to a radical change in the practice of medicine and many other inventions and discoveries of vast importance to the scientist, the agriculturist, the mechanic and the general public. The physician has found the improvement in the microscope, and its introduction into his practice of great utility in bringing into view and assisting him in studying the habits of the various spurea found in the human system (that have heretofore been the occult source of disease) and thereby enabling him to more aptly apply the appropriate remedies, and abridge the suffering of his patient.

In the surgical department, able minds have been at work, and much mechanical skill has been enlisted in developing new appliances, and modes of practice for remedying most of the serious accidents and organic lesions that befall our fellow beings. The telegraph, that wonder of the age, has annihilated both time and space, encircling the round world and bringing together the dwellers upon its surface, almost as one nation, kindred and people. The Heavens above, the earth below and the waters under the earth, have been made to yield their hidden mysteries, and to become as familiar household words to all, who will take the trouble to devote comparatively a few hours to study and observation. While all the nations of Europe have participated in the march of development and invention, America has not been behind the foremost in the paths of sciencc, as well as in industrial discoveries and inventions and among her citizens will be found men whose nanes will stand among the highest in in the temple of fame.

In our especial department of science, Mr. Editor, the progress has not been behind that of other professions, for dentistry in all its branches has been advanced to such an extent as to place the practice in a position to entitle it to a high degree of consideration with the public, and especially with the learned profession, the members of which formerly looked with much of contempt on these who followed the practice of dentistry, expressing their appreciation of its merits by characterizing the practice as a "trade," using the term in disparagement. But by a steady perseverance in well doing, in the part of those devoted to our profession, a large portion of these gentlemen engaged in the so-called learned profession have been led to hold in some little esteem, these individuals embarking in the once contemned practice, and likewise to accord to the collective devotees of dentistry, a decided recognition. A state of the case somewhat different from that of a few years since, when a "medical society" declined to receive a deputation from a dental convention, on a professional level, but fortunately for "the society" their action at that time, was expunged from their records at a succeeding meeting, and an expressive resolution was passed, ignoring their previous action.

However, there is yet evident an undercurrent of prejudice, although somewhat modified, and a homily could justly be read to a portion of that profesion from the text "Let him who is without sin (error) cast the first stone," let them correct their own lack of knowledge that is essential, for all are not as well posted as they might, could, would or should be, a clearer acquaintance with the injurious consequences to the general system from a diseased dental organization, might lead them many times to more happy results in the treatment of some of those neuralgic cases that are often found to baffle their labors. Dr. Rush claimed that particular attention to the condition of the teeth is of essential importance in the treatment of all diseases of a neuralgic type. While not claiming the present high opinion as essential to the life of our profession, still all engaged in it must feel gratified at its march upward and onward, in comparatively a few years and also, that its future is full of high promise for the comfort and health of our fellow beings. The assertion is evident from the fact that in days past the maladies attendant on the teeth and jaw required, in their latter and more complicated stages, the united aid of the surgeon and physician, yet they strictly in their earlier forms belonged to the practice of dentistry, and might not have required the assistance of the former if the latter had been afforded at the proper time, and by men who were qualified in the profession.

The advantages afforded by the dental colleges of the present day, is fast removing the necessity of calling in aid the general surgeon, for the professorships of these institutions are filled by men of decided talent, whose teachings have, to a large extent, blotted out that class of dentists, who possessed no proper understanding of the treatmant of these more complicated cases of disease of the teeth and jaws, and the pathological conditions of the parts involved in them. Now these decidedly useful and essential organs, the teeth, are being treated as living bodies, in contra-distinction of the inorganic theory held in past years, and being so highly endowed with vitality, as to be denominated "the fingers of the mouth."

To one who has, to some extent, been acquainted with the practice of dentisitry, for the past sixty years, and actually engaged in practice for nearly fifty years the improvements are indeed vast, and decidedly in advance of the condition of practice at the time when a Boston dentist who had accumulated a large fortune in practice, stated that it was entirely useless to attempt to fill the incisors when decayed on their anterior surfaces, as it was impossible to make them stick. There is no risk in the assertion that the *ne plus ultra* of operative dentistry has not been reached.

However the various complications at present so extensively in use by the profession for filling teeth, must strike many a timid patient with some degree of apprehension, for the display is indeed formidable. In case of caries in the lateral surface of a molar tooth, a separation is required between them, to obtain the desired end, the Electric or Morrison Engine, with its circular files or disks, is brought into requisition (accompanied by the cold water tank to keep the apparatus below the igniting point) when the required space is obtained then machinery for removing the decay and shaping the cavity, is used. That end accomplished and the gold made ready at hand, the "elastic profanity," the India rubber dam, is stretched over the necks of the teeth and gums and sustained in place by a cord tied above the necks of the teeth or the steel clamps are used, and these last are manipulated by the clamp forceps. When these appliances are attended to, the automatic mallet, a mallet and hand plugger with the striker, (for it takes two if not three to make a bargain in this case,) are ready the performance commences, and after a labor of two, four or eight hours, the cavity is filled to repletion forming a contour filling. The next step is to file down the gold, and polish; here again machinery comes into play; with this additional labor the operation is finished to the satisfaction of the operator, and the decided relief of the patient.

It would appear that one-half this labor has been bestowed in order to make a contour filling that will show for the noteriety of the operator, a beautiful piece of jeweler's work for all the superabundance of gold deposited around the cavity, after that is solidly fitted to its very edges, is surely of no use in preventing the teeth from farther decay; then wherein consists the utility of this style of work? where especially is the utility of it in an incisor, the third or half of the crown of which has been broken or decayed away longitudinally? you restore the lost substance with gold, do you make a natural looking tooth of it? surely not, and when two or four of the upper incisors are in a corresponding condition and filled in like manner, has the natural expression of the mouth in speaking been improved? far from it, for the view resembles more one obtained by casting a glance into a jeweler's show case, where you behold a display of jewels, dumb jewels, though each fail to speak in articulate words, they reflect the workman's ability in this branch of mechanics, but they do not, I think, speak for the process, as a saving one for decayed teeth, but it adds to the exchecquer of the operator, and gives him a notoriety with those who may not be well posted in the radical pre-requisites for filling, in order to save a tooth from further decay. A dimond in these anterior contour fillings would add to the brilliancy of the work, and answer equally well for protecting the teeth from further injury. The process appears to be a renewal in part of the old Roman practice of inserting on the roots crowns of gold, a practice in vogue in the days of old, but this was abandoned for the more reasonable one, so far as appearance is considered, of inserting crowns of bone.

I can not assent to the practice as adding to the beauty of the mouth, or for the salvation of a decayed tooth. After the gold is once firmly packed and the cavity filled to its edges, all the surplus is unnecesary labor to the operator, and a weariness to his patient, without an adequate return to the latter, although the former may reap some advantage in the higher figure charged, and in more extensively advertising the work of his hands. I am in the way of seeing every day a gentleman, in whose mouth are some eight fillings in the mesial and distal sides of the suparior incisors, that were put in over fifty years ago, by Dr. C. Stow Brewster, under the old system of practice, and the work has to-day the appearance of lasting another fifty years. The fillings are flush with the edges of the enamel, the gold of course solidly packed, well polished, soft gold and done with hand (ordinary) instruments. And it will be found that this is not the only instance of fillings remaining perfect for that number of years that have been put in by Dr. Brewster, and others of the old operators. At the risk of the charge of egotism, I will state here, that within the year past, I have met with a lady now a grandmother, whom when in her seventeenth year I filled ten or twelve teeth, and she stated that the majority of the fillings were then good. I had not seen her since operating on her, about thirty-seven years before. I wish I could express as much satisfaction in regard to all the work I have done, both before and since, no doubt I have done my share of bad. The devotees to the new system are too sweeping in their condemnation of the old practice, when they assert that a substantial filling can not be put into a tooth, unless a particular kind of foil is used, and with hand or automatic mallet, and all the new appliances, as some operators have asserted holding the new mode up as the only honest course to pursue in filling. To yield the point to them, is to acknowledge a false practice, in all the work done by gentlemen who have stood at the head of the profession, and whose operations rebut such assertions.

Some portions of the new appliances are no doubt of decided utility in filling the lower teeth, as well as others, the India rubber or coffer dam, seems essential in keeping both the cavity and the gold free from dampness, and the discovery is a rare gift to the profession, entitling the discoverer to the sincere thanks of both operator and patient, for his generosity in not securing for his discovery the protection of a patent, as many in the profession have done for discoveries of far less value, than "elastic profanity." Dr. Barnum has been in the receipt of some tangible rewards for his donation to the profession, and the gift has "damned him to everlasting fame."

My remarks may be regarded as decidedly hetrodox, and meet with condemnation by many of the profession or they may pass them by as the idle wind, and the writer placed among the "old fogies. I am indifferent as to the issue of opinion. From articles in dental periodicals, I learned that operators who were carried away with the new idea of filling have returned to their first love, and ignored the necessity of contour fillings as a means of salvation, either for themselves or their patients' teeth, and satisfied with fillings that are solid and flush with the mouth of the cavity exempting their patients from the enormous charges, attendant in making their mouths a store house for a surplus of gold foil or pellets and as a consequence a perambulating advertisement for the benefit of the operator, As an instance of enormous charges I have just observed in a New York paper, the charge of Dr. Atkinson, of one thousand and twenty-five dollars for filling four teeth for an actress.* My dull comprehension can not conceive of any teeth that would be worth that sum, even if the entire four, were composed of gold, with a respectable display of dimonds set in them, and the charge does appear enormous. Yet it is not up to the high figure Dr. A. has charged and collected. I think the workman is in all cases worthy of his hire, and those who are in the habit of charging thereby, may with justice claim that it is the patients' duty to first inquire the charge for filling, before submitting their dental organs to the manipulation of Dr. Atkinson or any other operator.

*There were six teeth filled. Ed.

